# B-Type Natriuretic Peptide Concentrations, COVID-19 Severity, and Mortality: A Systematic Review and Meta-Analysis With Meta-Regression

**DOI:** 10.3389/fcvm.2021.690790

**Published:** 2021-06-24

**Authors:** Angelo Zinellu, Salvatore Sotgia, Ciriaco Carru, Arduino A. Mangoni

**Affiliations:** ^1^Department of Biomedical Sciences, University of Sassari, Sassari, Italy; ^2^Quality Control Unit, University Hospital of Sassari, Sassari, Italy; ^3^Discipline of Clinical Pharmacology, College of Medicine and Public Health, Flinders University, Adelaide, SA, Australia; ^4^Department of Clinical Pharmacology, Flinders Medical Centre, Southern Adelaide Local Health Network, Adelaide, SA, Australia

**Keywords:** B-type natriuretic peptide, COVID-19, disease severity, mortality, biomarkers

## Abstract

Alterations in cardiac biomarkers have been reported in patients with coronavirus disease 2019 (COVID-19) in relation to disease severity and mortality. We conducted a systematic review and meta-analysis with meta-regression of studies reporting B-type natriuretic peptide (BNP) or N-terminal proBNP (NT-proBNP) plasma concentrations in COVID-19. We searched PubMed, Web of Science, and Scopus, between January 2020 and 2021, for studies reporting BNP/NT-proBNP concentrations, measures of COVID-19 severity, and survival status (PROSPERO registration number: CRD42021239190). Forty-four studies in 18,856 COVID-19 patients were included in the meta-analysis and meta-regression. In pooled results, BNP/NT-proBNP concentrations were significantly higher in patients with high severity or non-survivor status when compared to patients with low severity or survivor status during follow up (SMD = 1.07, 95% CI: 0.89–1.24, and *p* < 0.001). We observed extreme between-study heterogeneity (*I*^2^ = 93.9%, *p* < 0.001). In sensitivity analysis, the magnitude and the direction of the effect size were not substantially modified after sequentially removing individual studies and re-assessing the pooled estimates, (effect size range, 0.99 – 1.10). No publication bias was observed with the Begg's (*p* = 0.26) and Egger's (*p* = 0.40) *t*-tests. In meta-regression analysis, the SMD was significantly and positively associated with D-dimer (*t* = 2.22, *p* = 0.03), myoglobin (*t* = 2.40, *p* = 0.04), LDH (*t* = 2.38, *p* = 0.02), and procalcitonin (*t* = 2.56, *p* = 0.01) concentrations. Therefore, higher BNP/NT-proBNP plasma concentrations were significantly associated with severe disease and mortality in COVID-19 patients.

## Introduction

A significant number of clinical and demographic factors have been studied in patients with coronavirus disease 19 (COVID-19) in regard to their association with specific clinical presentations and measures of clinical severity (1, 2). The evidence of an excessive activation of inflammatory and immunomodulating pathways in patients with the more severe forms of the disease, typically characterized by the development of respiratory failure with or without multi-organ dysfunction, have prompted the search for specific biomarkers of inflammation and immuno-activation in order to develop better predictive models to assist with management (3). The increasing evidence of significant alterations of different organs and/or systems in patients with COVID-19 has also led to the investigation of the predictive capacity of additional, organ-specific, biomarkers. For example, the presence of myocardial injury, associated with several cardiac manifestations, including myocarditis, acute coronary syndrome, and arrhythmias, has been well-documented in COVID-19 patients with or without pre-existing cardiovascular history (4). Notably, cardiac abnormalities in this group are independently associated with an increased risk of mortality (5). While the exact mechanisms involved in the onset and progression of COVID-19 related myocardial injury remain to be elucidated, several circulating markers of myocardial damage, particularly creatine kinase (CK), and troponin, are being increasingly studied in terms of their predictive capacity (6). Another cardiac complication, heart failure, has been observed in about a quarter of patients with COVID-19 and has been associated with an increased risk of adverse outcomes (7, 8). The active peptide B-type natriuretic peptide (BNP) and the inactive peptide N-terminal proBNP (NT-proBNP) are both derived from the human BNP precursor proBNP in the ventricular myocytes. The increased secretion of BNP and NT-proBNP from the heart, in response to high ventricular filling pressures, is routinely used as a diagnostic and prognostic marker in heart failure and, by some, as a marker of the size or severity of ischaemic insults (9–11). However, its biological and clinical role in patients with COVID-19 is not well-established. We addressed this issue by conducting a systematic review and meta-analysis with meta-regression of studies reporting plasma BNP or NT-proBNP concentrations in COVID-19 patients with different disease severity, based on clinical guidelines or need for hospitalization, mechanical ventilation, or transfer to the intensive care unit (ICU), and clinical outcomes, particularly survival status during follow up.

## Materials and Methods

### Search Strategy, Eligibility Criteria, and Study Selection

We conducted a systematic literature search, using the terms “brain natriuretic peptide” or “BNP” or “NT-proBNP” or “N-terminal pro-brain natriuretic peptide” and “coronavirus disease 19” or “COVID-19,” in PubMed, Web of Science and Scopus, from January 2020 to January 2021, to identify peer-reviewed studies reporting BNP/NT-proBNP concentrations in COVID-19 patients according to disease severity and/or mortality. We accessed the references of the retrieved articles to identify additional studies. Eligibility criteria were (a) reporting continuous data on plasma BNP/NT-proBNP concentrations in COVID-19 patients, (b) investigating COVID-19 patients with different disease severity or survival status during follow up, (c) adult patients, (d) English language, (e) >10 patients, and (f) full-text available. Two investigators independently screened individual abstracts. If relevant, they independently reviewed the full articles (PROSPERO registration number: CRD42021239190). We used the Newcastle-Ottawa scale to assess study quality, with a score ≥6 indicating high quality (12).

### Statistical Analysis

We calculated standardized mean differences (SMD) and 95% confidence intervals (CIs) in BNP/NT-proBNP concentrations between COVID-19 patients with low vs. high severity or survivor vs. non-survivor status. A *p* < 0.05 was considered statistically significant. When studies reported medians and interquartile ranges (IQR) the corresponding means and standard deviations were estimated (13). We assessed between-study heterogeneity in SMD values using the Q-statistic (significance level at *p* < 0.10). Inconsistency across studies was evaluated using the *I*^2^ statistic, where *I*^2^ <25% indicated no heterogeneity, between 25 and 50% moderate heterogeneity, between 50 and 75% large heterogeneity, and >75% extreme heterogeneity (14, 15). Random-effect models were used to calculate the pooled SMD and 95% CIs if significant heterogeneity was present. In sensitivity analyses, the influence of individual studies on the overall effect size was assessed using the leave-one-out method (16). The presence of publication bias was assessed using the Begg's and the Egger's test, at the *p* < 0.05 level of significance (17, 18), and the Duval and Tweedie “trim and fill” procedure (19). To identify factors contributing to the between-study variance, we investigated the effects of several biologically and/or clinically plausible factors on the SMD by univariate meta-regression analysis. These factors included age, gender, clinical endpoint, study design (retrospective or prospective), geographical area where the study was conducted, aspartate aminotransferase (AST), alanine aminotransferase (ALT), D-dimer, serum creatinine, myoglobin, troponin, CK, albumin, ferritin, lactate dehydrogenase (LDH), procalcitonin, C-reactive protein (CRP), white blood cell count (WBC), diabetes, hypertension and cardiovascular disease. Statistical analyses were performed using Stata 14 (STATA Corp., College Station, TX, USA). The study was fully compliant with the PRISMA statement (20).

## Results

### Literature Search and Study Selection

**Figure 1:**
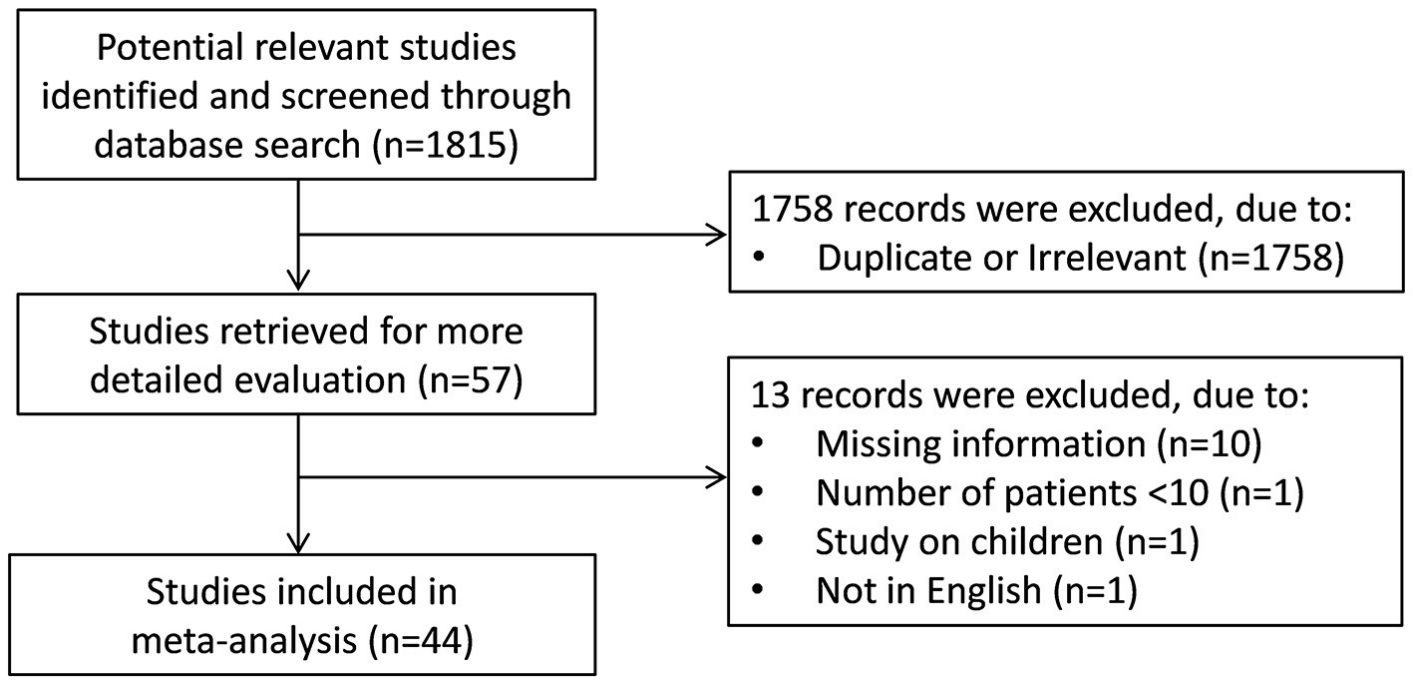
Study selection flow chart.

We initially identified 1,815 studies. A total of 1,758 studies were excluded after the first screening because they were duplicates or irrelevant. Following full-text revision of the remaining 57 articles, 13 were further excluded because they did not meet the inclusion criteria. Thus, 44 studies in 18,856 COVID-19 patients, 14,569 (53% males, mean age 48 years) with low severity or survivor status and 4,287 (59% males, mean age 61 years) with high severity or non-survivor status, were included in the final analysis (Figure 1 and Table 1) (7, 21–63). Thirty-two studies were conducted in Asia (7, 22, 24, 26, 27, 29, 31–33, 35, 38–46, 48, 50, 51, 53–55, 57–63), eight in Europe (25, 28, 30, 34, 47, 49, 52, 56), three in America (23, 36, 37), and one in Africa (21). Thirty-two studies were retrospective (7, 21–23, 27, 29, 31–39, 41–45, 48, 50, 51, 53, 55, 57–63), eight prospective (24, 30, 46, 47, 49, 52, 54, 56), whereas the remaining four did not report the study design (25, 26, 28, 40). Clinical endpoints included disease severity based on current clinical guidelines in 15 studies (21, 24, 33, 39–41, 43, 48, 54, 55, 57–59, 62, 63), hospitalization in one (37), ICU transfer in three (32, 44, 49), or need for mechanical ventilation in one (23), and survival status in 24 studies (7, 22, 25–31, 34–36, 38, 42, 45–47, 50–53, 56, 60, 61). Sixteen studies reported plasma BNP concentrations (23, 24, 26, 33, 35–37, 45, 48, 50, 51, 54, 56–58, 62), whereas the remaining 28 reported plasma NT-proBNP concentrations (7, 21, 22, 25, 27–32, 34, 38–44, 46, 47, 49, 52, 53, 55, 59–61, 63). Only one study reported cumulative 7-day mean plasma NT-proBNP concentrations (34), whereas another reported BNP concentrations on initial presentation to the emergency department (37). The remaining 42 studies reported BNP or NT-proBNP concentrations within the first 24–48 h from admission.

**Table 1:** Characteristics of the selected studies.

					**Low severity or survivor**				**High severity or non-survivor**			
**References**	**Country**	**Study design**	**Endpoint**	**NOS (stars)**	***n***	**Age (Years)**	**Gender (M/F)**	**BNP pg/mL (Mean ± SD)**	***n***	**Age (Years)**	**Gender (M/F)**	**BNP pg/mL (Mean ± SD)**
Abdeladim et al. (21)	Morocco	R	Disease severity	6	39	50	11/28	81 ± 50^*^	34	61	12/22	2,982 ± 3,172^*^
Aladag et al. (22)	Turkey	R	Survival status	7	35	68	22/13	3,318 ± 5,054^*^	15	68	6/9	15,511 ± 13,638^*^
Almeida Junior et al. (23)	Brazil	R	Survival status or MV	7	139	64	86/53	73 ± 90	44	76	34/10	287 ± 485
Bao et al. (24)	China	P	Disease severity	5	129	NR	NR	11 ± 25	49	NR	NR	53 ± 85
Belarte-Tornero et al. (25)	Spain	NR	Survival status	7	82	77	45/37	518 ± 528^*^	47	86	18/29	5,192 ± 6,673^*^
Chen et al. (26)	China	NR	Survival status	8	1,651	57	781/870	67 ± 93	208	70	153/55	685 ± 987
Chen et al. (7)	China	R	Survival status	6	161	51	88/73	92 ± 122^*^	113	68	83/30	1,002 ± 1,058^*^
Chen et al. (27)	China	R	Survival status	8	53	64	27/26	336 ± 298^*^	20	69	15/5	840 ± 898^*^
Ciceri et al. (28)	Italy	NR	Survival status	8	291	62	207/84	206 ± 259^*^	95	76	70/25	1,583 ± 2,176^*^
Cui et al. (29)	China	R	Survival status	8	699	61	353/346	153 ± 158^*^	137	70	86/51	1,244 ± 1,649^*^
D'Alto et al. (30)	Italy	P	Survival status	8	69	62	53/16	686 ± 1,224^*^	25	68	17/8	3,375 ± 3,891^*^
Deng et al. (31)	China	R	Survival status	8	212	63	97/115	227 ± 293^*^	52	75	33/19	1,248 ± 1,478^*^
Du et al. (32)	China	R	Transfer to ICU	6	58	73	31/27	852 ± 620^*^	51	68	40/11	564 ± 654^*^
Feng et al. (33)	China	R	Disease severity	6	352	51	190/162	41 ± 64	124	60	81/43	65 ± 67
Ferrari et al. (34)	Italy	R	Survival status	6	40	60	27/13	690 ± 1,075^*^	42	74	30/12	6,296 ± 17,528^*^
Gan et al. (35)	China	R	Survival status	8	56	62	30/26	1,653 ± 289	39	70	28/11	1,848 ± 784
Gavin et al. (36)	USA	R	Survival status	6	118	57	58/60	160 ± 51	18	73	11/7	587 ± 184
Gottlieb et al. (37)	USA	R	Hospitalization	8	7,190	38	3,935/3,255	33 ± 35	1,483	58	792/691	73 ± 74
Guo et al. (38)	China	R	Survival status	8	28	59	NR	1,741 ± 2,363^*^	46	72	NR	3,544 ± 7,998^*^
Han et al. (39)	China	R	Disease severity	6	198	59	127/71	145 ± 169^*^	75	59	26/49	624 ± 1,027^*^
He et al. (40)	China	NR	Disease severity	6	32	42	15/17	42 ± 66^*^	21	57	13/8	822 ± 1,100^*^
He et al. (41)	China	R	Disease severity	8	530	60	241/289	83 ± 95^*^	501	66	297/204	381 ± 498^*^
Hui et al. (42)	China	R	Survival status	8	65	55	42/23	176 ± 178^*^	47	66	29/18	1,631 ± 2,453^*^
Koc et al. (43)	Turkey	R	Disease severity	6	60	65	37/23	53 ± 35^*^	30	61	20/10	267 ± 339^*^
Li et al. (44)	China	R	Transfer to ICU	8	312	49	131/181	100 ± 129^*^	211	62	119/92	103 ± 132^*^
Li et al. (45)	China	R	Survival status	6	60	62	33/27	327 ± 455	14	71	11/3	854 ± 849
Liu et al. (46)	China	P	Survival status	8	21	64	15/6	1,859 ± 2,599^*^	22	65	7/15	7,530 ± 8,820^*^
Lorente et al. (47)	Soain	P	Survival status	7	118	64	53/65	538 ± 789^*^	25	71	7/18	3,370 ± 4,218^*^
Ma et al. (48)	China	R	Disease severity	6	429	42	230/199	180 ± 273	94	50	59/35	663 ± 641
Myhre et al. (49)	Norway	P	Survival status or transfer to ICU	8	88	58	46/42	126 ± 170^*^	35	64	25/10	186 ± 181^*^
Pan et al. (50)	China	R	Survival status	8	35	65	18/17	63 ± 73	89	69	67/22	107 ± 113
Qin et al. (51)	China	R	Survival status	8	239	63	113/126	93 ± 102	23	69	10/13	499 ± 468
Rath et al. (52)	Germany	P	Survival status	7	107	67	65/42	808 ± 1,320^*^	16	73	12/4	3,376 ± 5,410^*^
Sun et al. (53)	China	R	Survival status	8	123	67	51/72	2 ± 2^*^	121	72	82/39	13 ± 16^*^
Sun et al. (54)	China	P	Disease severity	7	49	52	26/23	9 ± 6	50	71	34/16	163 ± 232
Tao et al. (55)	China	R	Disease severity	7	202	54	72/130	198 ± 352^*^	20	65	8/12	811 ± 1,367^*^
Vrillon et al. (56)	France	P	Survival status	8	54	90	19/35	184 ± 242	22	90	15/7	367 ± 371
Wang et al. (57)	China	R	Disease severity	6	72	NR	24/48	48 ± 46	38	NR	24/14	206 ± 228
Xie et al. (58)	China	R	Disease severity	7	38	61	26/12	29 ± 29	24	72	12/12	97 ± 106
Yang et al. (59)	China	R	Disease severity	6	99	44	49/50	1,705 ± 2,326^*^	15	60	7/8	243 ± 165^*^
Yu et al. (60)	China	R	Survival status	8	123	80	46/77	299 ± 287^*^	18	84	11/7	2 ± 349,941^*^
Zhang et al. (61)	China	R	Survival status	6	62	60	35/27	310 ± 441^*^	36	71	23/13	3,200 ± 5,144^*^
Zhao et al. (62)	China	R	Disease severity	8	19	49	7/12	97 ± 115	31	60	23/8	703 ± 641
Zheng et al. (63)	China	R	Disease severity	6	32	44	NR	67 ± 91^*^	67	64	NR	1,086 ± 3,217^*^

*ICU, intensive care unit; MV, mechanical ventilation; NOS, Newcastle-Ottawa quality assessment scale for case-control studies; NR, not reported; P, prospective; R, retrospective*;

^*^ *, NT-proBNP*.

### Meta-Analysis

**Figure 2:**
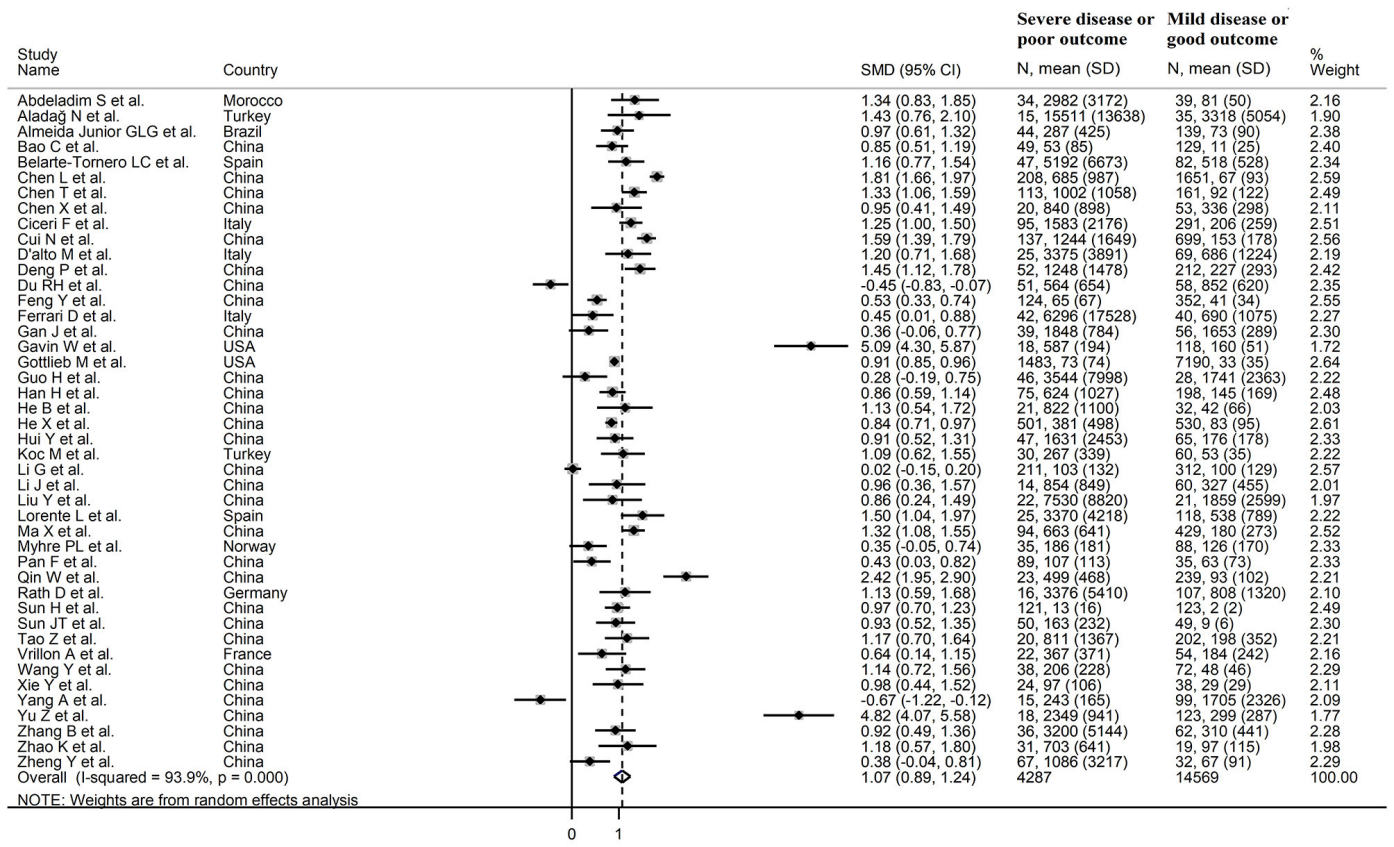
Forest plot of studies reporting BNP concentrations in patients with COVID-19.

The overall SMD in BNP/NT-proBNP concentrations between COVID-19 patients with low vs. high severity or survivor vs. non-survivor status is shown in Figure 2. In two studies, patients with high severity or non-survivor status had significantly lower BNP/NT-proBNP concentrations when compared to those with low severity or survivor status (mean difference range, −0.45 to −0.67) (31, 59). By contrast, in the remaining studies BNP/NT-proBNP concentrations were lower in patients with low severity or survivor status (mean difference range, 0.02 – 5.09), with a non-significant difference in five studies (35, 38, 44, 50, 63). Pooled results confirmed that BNP/NT-proBNP concentrations were significantly higher in patients with severe disease or non-survivor status (SMD = 1.07, 95% CI: 0.89 – 1.24, and *p* < 0.001; Figure 2). There was extreme between-study heterogeneity (*I*^2^ = 93.9%, *p* < 0.001). BNP/NT-proBNP concentrations remained significantly higher (SMD = 1.06, 95% CI: 0.86 – 1.26, and *p* < 0.001; *I*^2^ = 93.0%, *p* < 0.001) in patients with high severity or non-survivor status after excluding two relatively large studies, accounting for nearly 56% of the overall sample size (26, 37).

**Figure 3:**
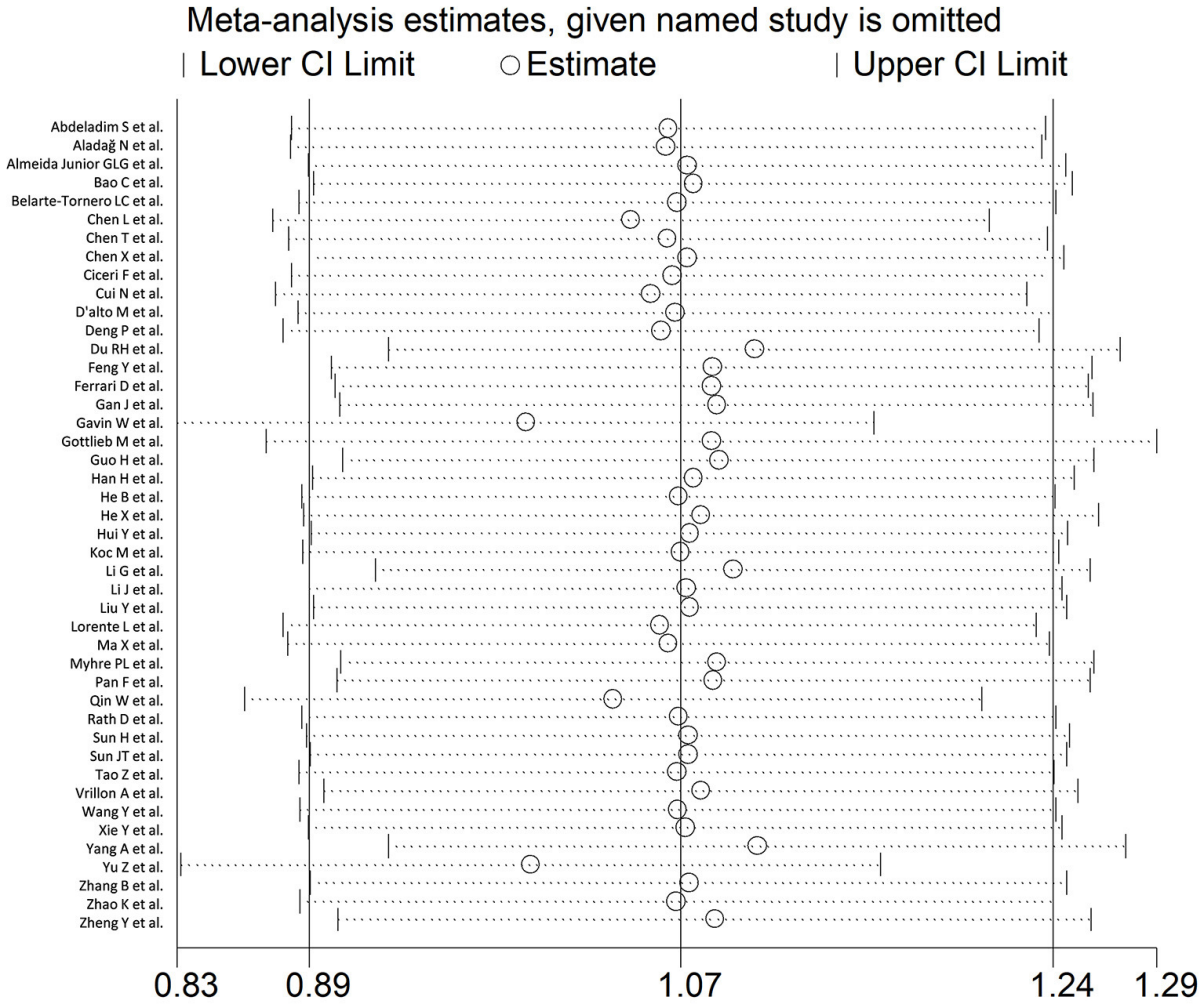
Sensitivity analysis of the association between BNP and COVID-19 disease, assessed by investigating the influence of individual studies on the overall standardized mean difference (SMD). The SMD and the 95% confidence intervals (CIs) are indicated by the middle and the lateral vertical axes, respectively. The pooled SMD and the 95% CIs are indicated by the hollow circles and the two ends of each broken line, respectively.

**Figure 4:**
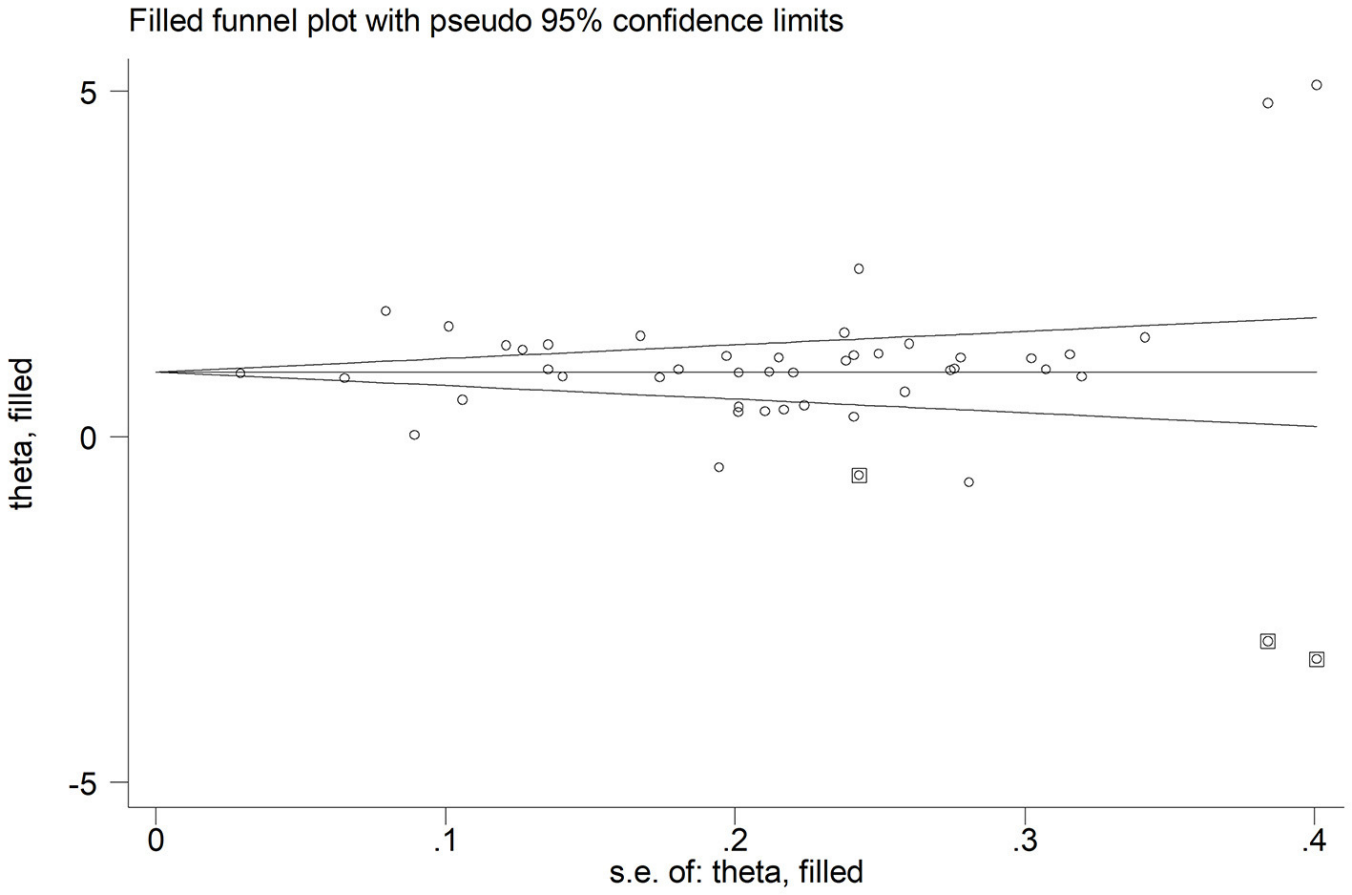
Funnel plot of studies investigating disease severity or survival status after trimming and filling. Enclosed and free circles indicate dummy and genuine studies, respectively.

In sensitivity analysis, the magnitude and the direction of the effect size were not substantially modified after sequentially removing each study and re-assessing the pooled estimates (effect size range, 0.99 – 1.10; Figure 3). No publication bias was observed with the Begg's (*p* = 0.26) and Egger's (*p* = 0.40) *t*-tests. However, using the trim-and-fill method, we identified three potential missing studies to be added to the left side of the funnel plot to ensure symmetry (Figure 4). The adjusted SMD, albeit attenuated, remained significant (SMD = 0.90, 95% CI: 0.70 – 1.09, and *p* < 0.001).

**Figure 5:**
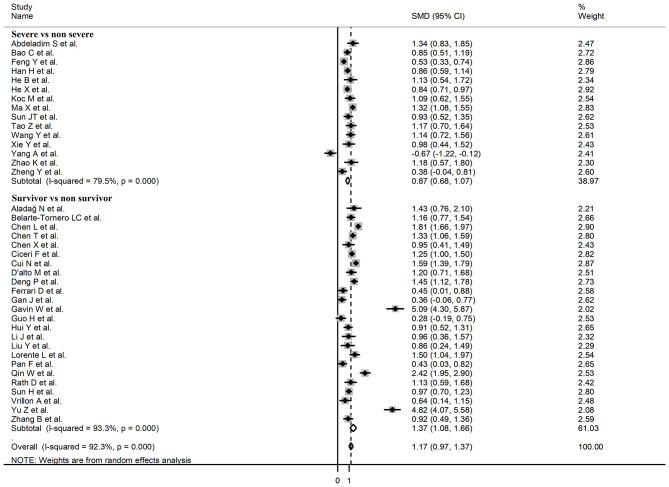
Forest plot of studies reporting BNP concentrations in patients with COVID-19 according to disease severity or survival status.

**Figure 6:**
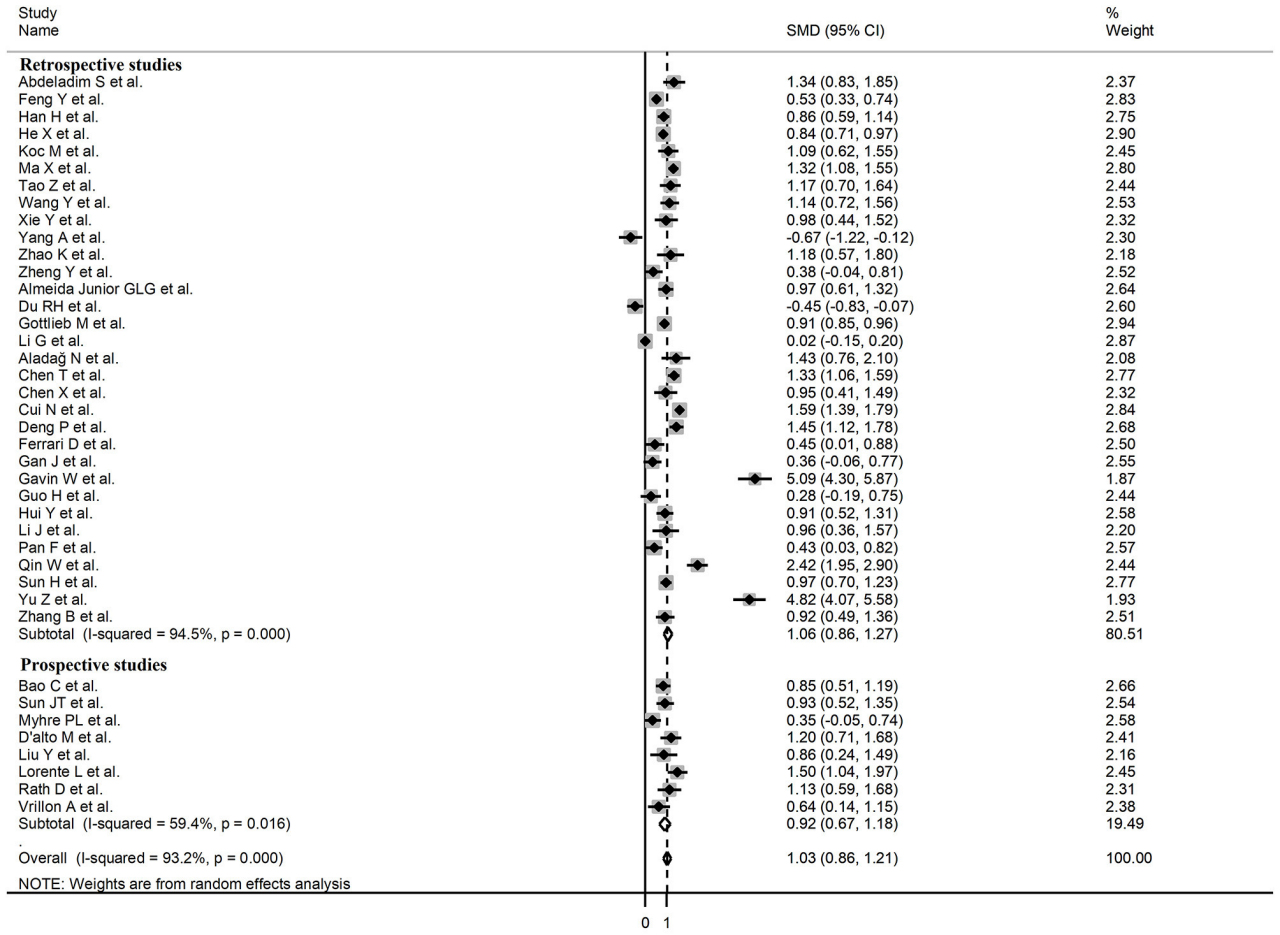
Forest plot of studies reporting BNP concentrations in patients with COVID-19 according to study design (retrospective or prospective).

**Figure 7:**
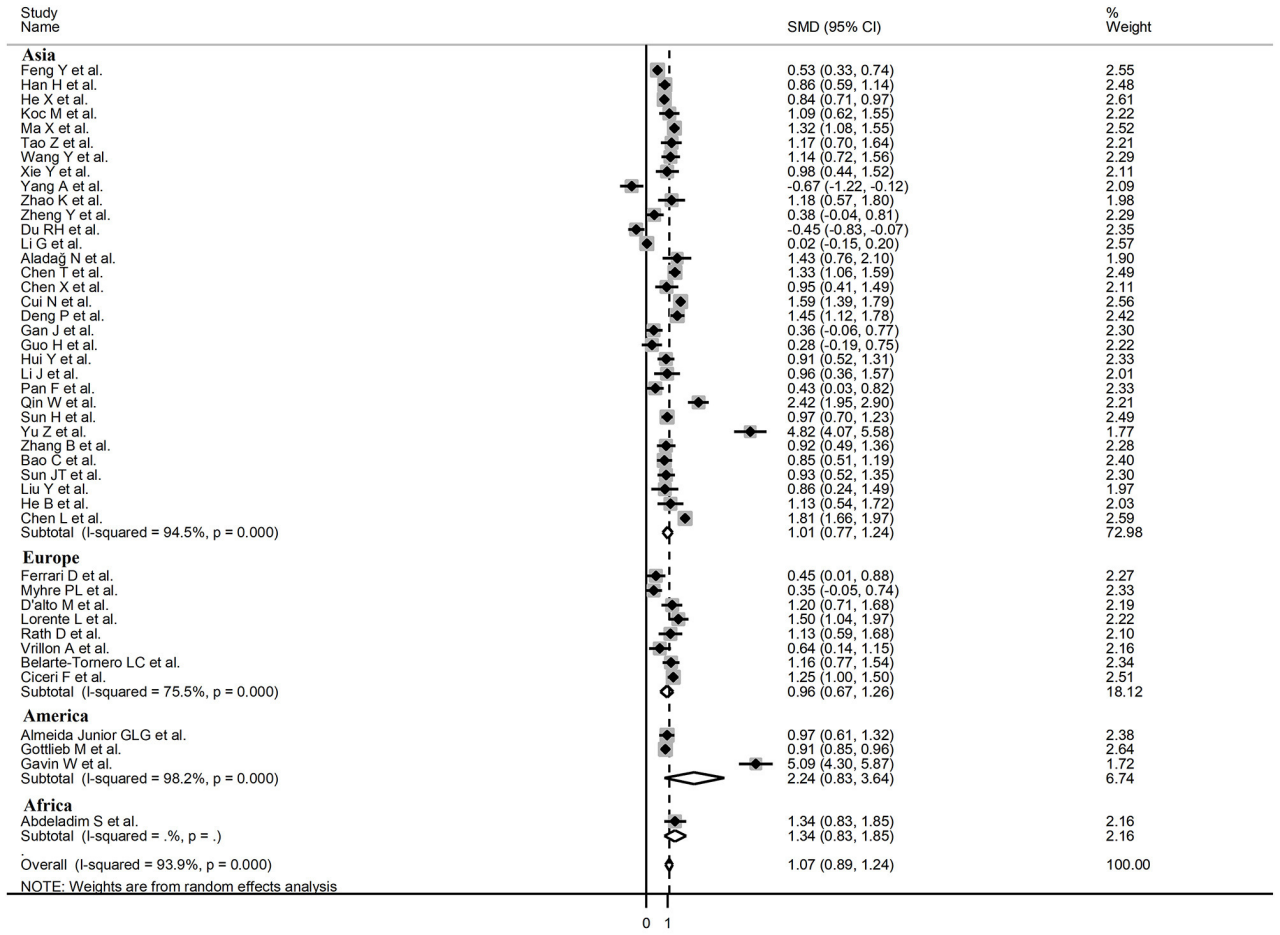
Forest plot of studies examining BNP concentrations in patients with COVID-19 according to the geographic area where the study was conducted.

**Figure 8:**
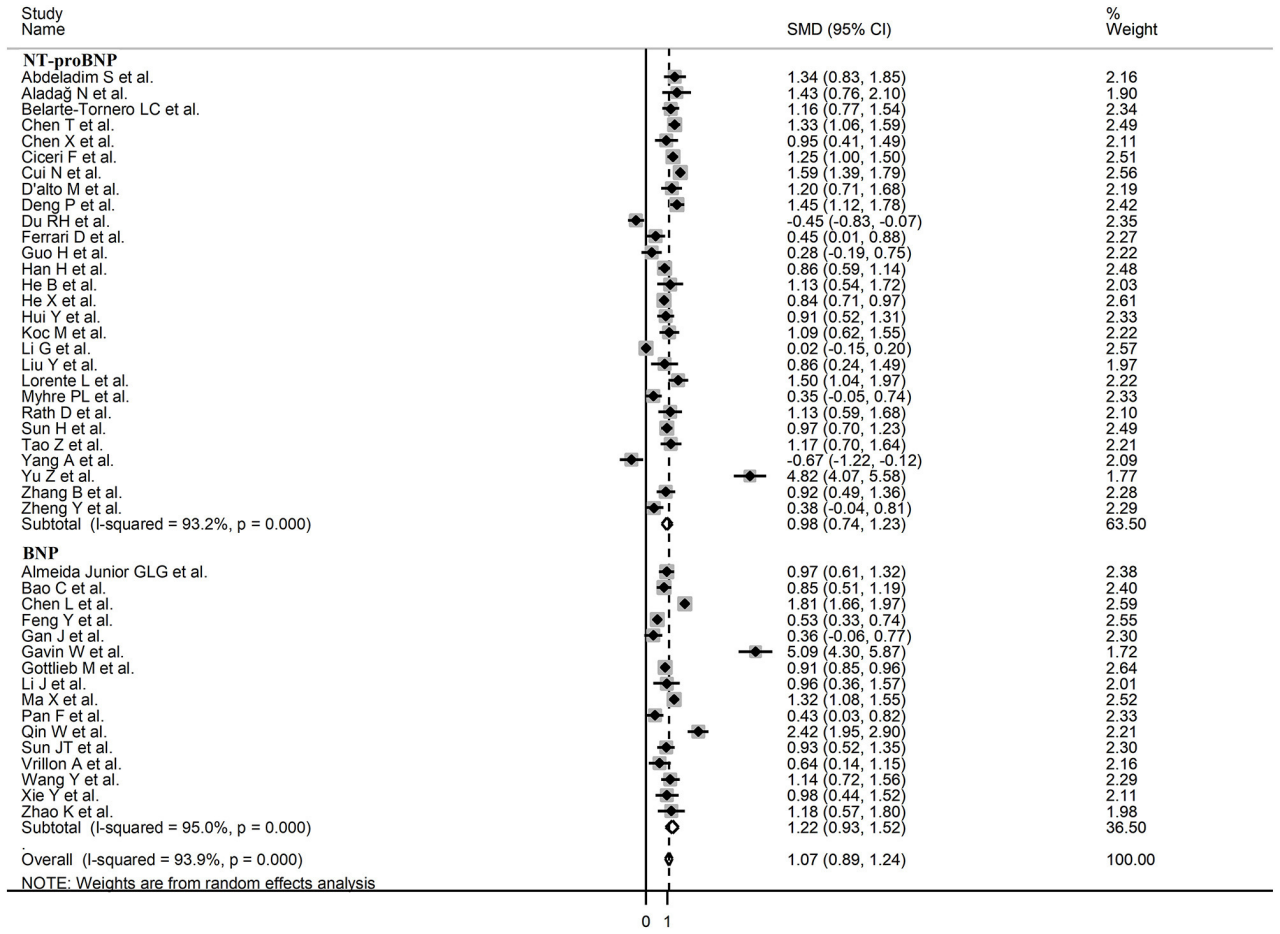
Forest plot of studies examining BNP concentrations in patients with COVID-19 according to whether plasma NT-proBNP or BNP was measured.

Sub-group analysis of the 42 studies reporting BNP/NT-proBNP concentrations on admission showed that the SMD remained significantly higher in patients with high severity or non-survivor status (SMD = 1.10, 95% CI: 0.88 – 1.31, and *p* < 0.001) with an extreme between-study variance (*I*^2^ = 94.1%, *p* < 0.001). Additionally, the pooled SMD value in studies assessing disease severity (SMD = 0.87, 95% CI: 0.68 – 1.07, and *p* < 0.001; *I*^2^ = 79.5, *p* < 0.001) was non-significantly lower than those investigating survivor status (SMD = 1.37, 95% CI: 1.08 – 1.66, *p* < 0.001; *I*^2^ = 92.3, *p* < 0.001; *t* = 1.63, *p* = 0.11; Figure 5). Similarly, non-significantly higher SMD values were observed in retrospective (SMD = 1.06, 95% CI: 0.86 – 1.27, *p* < 0.001; *I*^2^ = 94.5, *p* < 0.001) vs. prospective studies (SMD = 0.92, 95% CI: 0.67 – 1.18, *p* < 0.001; *I*^2^ = 59.4, *p* = 0.016; *t* = −0.41, *p* = 0.69; Figure 6). The pooled SMD value in European studies (SMD = 0.96, 95% CI: 0.67 – 1.26, *p* < 0.002; *I*^2^ = 75.5%, *p* < 0.001) was non-significantly lower than that observed in Asian (SMD = 1.01, 95% CI: 0.77 – 1.24, *p* < 0.001; *I*^2^ = 94.5%, *p* < 0.001) and American studies (SMD = 2.24, 95% CI: 0.83 – 3.64, *p* < 0.001; *I*^2^ = 98.2%, *p* < 0.001; *t* = 1.36, *p* = 0.18; Figure 7). Finally, the pooled SMD value in studies reporting plasma NT-proBNP concentrations (SMD = 0.98, 95% CI: 0.74 – 1.23, *p* < 0.001; *I*^2^ = 93.2%, *p* < 0.001) was non-significantly lower than that observed in studies reporting plasma BNP concentrations (SMD = 1.22, 95% CI: 0.93 – 1.52, *p* < 0.001; *I*^2^ = 95.0%, *p* < 0.001; *t* = −0.85, *p* = 0.40; Figure 8). A relatively lower heterogeneity was observed in prospective (*I*^2^ = 59.4%) and European studies (*I*^2^ = 75.5%), and in those investigating disease severity (*I*^2^ = 79.5%).

### Meta-Regression

The D-dimer (*t* = 2.22, *p* = 0.03), myoglobin (*t* = 2.40, *p* = 0.04), LDH (*t* = 2.38, *p* = 0.02), and procalcitonin (*t* = 2.56, *p* = 0.01) concentrations were significantly and positively associated with the pooled SMD. By contrast, no significant correlations were observed between the SMD and age (*t* = −0.30, *p* = 0.76), gender (*t* = 0.26, *p* = 0.80), AST (*t* = 0.25, *p* = 0.81), ALT (*t* = −0.89, *p* = 0.38), creatinine (*t* = 0.93, *p* = 0.36), troponin (*t* = 0.18, *p* = 0.86), CK (*t* = 0.85, *p* = 0.41), albumin (*t* = 0.70, *p* = 0.49), ferritin (*t* = −1.29, *p* = 0.22), CRP (*t* = 0.96, *p* = 0.34), WBC (*t* = 0.08, *p* = 0.94), diabetes (*t* = −0.59, *p* = 0.56), hypertension (*t* = −0.01, *p* = 0.99), and cardiovascular disease (*t* = −0.53, *p* = 0.60).

## Discussion

In our study, plasma concentrations of BNP and NT-proBNP, generally measured within the first 24–48 h from admission, were significantly higher in COVID-19 patients with severe disease, based on clinical assessment or the need for hospitalization, mechanical ventilation, or ICU transfer, and in those who did not survive when compared to patients with mild disease or who survived during follow up. The observed SMD values for combined natriuretic peptide concentrations or BNP and NT-proBNP separately, 1.07, 1.22, and 0.98, respectively, suggest a biologically and clinically significant effect size (64). Although between-study heterogeneity was extreme, in sensitivity analysis the effect size was not influenced when individual studies were sequentially removed. The Begg's and Egger's *t*-tests did not show any evidence of publication bias. In meta-regression analysis, significant associations were observed between the SMD value and D-dimer, myoglobin, LDH, and procalcitonin, but not with age, gender, AST, ALT, creatinine, troponin, CK, albumin, ferritin, CRP, WBC, diabetes, hypertension, or cardiovascular disease.

Differently from the inactive NT-proBNP, the BNP exerts significant biological effects through its binding to the guanylyl cyclase-coupled natriuretic receptors A and B. The consequent increase in cyclic guanosine monophosphate causes vasodilatation, diuresis, natriuresis, inhibition of the renin-angiotensin-aldosterone system, inhibition of fibrosis, hypertrophy, cell apoptosis and inflammation, including suppression of superoxide generation by neutrophils, and improvement in myocardial relaxation (10). Notably, there is no evidence of significant associations between BNP and cyclic guanosine monophosphate concentrations in human studies. Furthermore, specific BNP-mediated protective effects, particularly the suppression of neutrophil-mediated generation of superoxide via nicotinamide adenine dinucleotide phosphate oxidase, are impaired in the context of acute heart failure, even in the presence of increased BNP concentrations (65). Whilst such effects are partially restored with pharmacological treatment, the failure of BNP-related suppression of superoxide release might lead to sustained tissue inflammation in heart failure, with or without concomitant COVID-19. There are other differences between the BNP and the NT-proBNP, with the latter being characterized by a higher molecular mass, a longer half-life (>60 vs. 15–20 min), a higher degree of *in vivo* glycosylation, and a lower degree of intra-individual biological variation (66). The better analytical characteristics of the available immunoassay methods for the measurement of NT-proBNP concentrations, when compared to those for the assessment of the BNP, have prompted some experts to advocate the measurement and monitoring of NT-proBNP concentrations as the best strategy for the management of patients with heart failure (66). These issues notwithstanding, in our meta-analysis the studies reporting BNP vs. NT-proBNP plasma concentrations had similar SMD values and degrees of heterogeneity.

The significant association observed between plasma BNP/NT-proBNP concentrations, disease severity and mortality in patients with COVID-19 is likely to reflect the presence of heart failure and its adverse sequelae in this group. In this context, these routine and relatively inexpensive biomarkers might assist the clinician with the early diagnosis of cardiac dysfunction and the prompt initiation of appropriate pharmacological and non-pharmacological therapies (67). Further research is warranted to determine whether the assessment of BNP/NT-proBNP might also be incorporated into predictive tools that are specifically developed and validated in COVID-19 patients. The reported associations, in meta-regression analysis, between the SMD of BNP/NT-proBNP and D-dimer, myoglobin, LDH, and pro-calcitonin suggests that the effect size is particularly correlated with markers of pro-coagulant activity, skeletal muscle and other tissue damage, and severe sepsis, respectively. Notably, these markers have, in turn, been shown to have significant associations with COVID-19 severity and outcomes (68–70). By contrast, the lack of associations observed with other cardiac biomarkers, e.g., troponin and CK, suggests that the measurement of BNP/NT-proBNP may provide complementary, rather than redundant, information regarding the presence of cardiac abnormalities in patients with COVID-19.

The extreme between-study heterogeneity represents a limitation of our study. However, we did not observe significant publication bias and the overall effect size was not substantially affected in sensitivity analyses. The lack of significant associations between the SMD and several patient and study characteristics, except for D-dimer, myoglobin, LDH, and procalcitonin concentrations, suggests that other unreported factors might have contributed to the observed heterogeneity. Such factors may include the relationship between the SMD values and the presence of new onset vs. acute on chronic heart failure and the information regarding the specific analytical methods used for the determination of BNP and NT-proBNP plasma concentrations (66). In this context, the lack of available information on indexes of left ventricular function prevented the conduct of further meta-regression analyses of the association between such indexes and the SMD values. Furthermore, virtually all selected studies reported isolated, rather than serial, measurements of natriuretic peptide shortly after hospital admission. This issue is particularly important as the routine monitoring of BNP/NT-proBNP concentrations has been shown to be beneficial in heart failure (71). Further studies should investigate the prognostic value of single vs. serial BNP/NT-proBNP measurements also in patients with COVID-19.

In conclusion, higher plasma concentrations of BNP or NT-proBNP are significantly associated with higher disease severity and increased mortality in COVID-19. Additional studies are required to determine whether these cardiac biomarkers can be incorporated into robust predictive tools that further assist with early management and monitoring in this patient group.

## Data Availability Statement

The original contributions presented in the study are included in the article/Supplementary Material, further inquiries can be directed to the corresponding author/s.

## Author Contributions

AZ: initial idea. AZ and SS: data collection and analysis. AZ, SS, CC, and AM: data interpretation and writing—review and editing. AM: writing—first draft. All authors contributed to the article and approved the submitted version.

## Conflict of Interest

The authors declare that the research was conducted in the absence of any commercial or financial relationships that could be construed as a potential conflict of interest.
